# Rapid Changes in Microbial Community Structures along a Meandering River

**DOI:** 10.3390/microorganisms8111631

**Published:** 2020-10-22

**Authors:** Perrine Cruaud, Adrien Vigneron, Caetano C. Dorea, Manuel J. Rodriguez, Steve J. Charette

**Affiliations:** 1 Institut de Biologie Intégrative et des Systèmes (IBIS), Université Laval, Laval, QC G1V 0A6, Canada; avignero@gmail.com (A.V.); Steve.Charette@bcm.ulaval.ca (S.J.C.); 2 Département de Biochimie, de Microbiologie et de Bio-Informatique, Faculté des Sciences et Génie, Université Laval, Laval, QC G1V 0A6, Canada; 3 Centre de Recherche en Aménagement et Développement (CRAD), Université Laval, Laval, QC G1V 0A6, Canada; Manuel.Rodriguez@esad.ulaval.ca; 4 Département de Biologie, Université Laval, Laval, QC G1V 0A6, Canada; 5 Centre d’Études Nordiques, Université Laval, Laval, QC G1V 0A6, Canada; 6 Department of Civil Engineering, University of Victoria, Victoria, BC V8P 5C2, Canada; caetanodorea@uvic.ca; 7 École Supérieure d’Aménagement du Territoire et de Développement Régional (ESAD), Université Laval, Laval, QC G1V 0A6, Canada; 8 Centre de Recherche de l’Institut Universitaire de Cardiologie et de Pneumologie de Québec, Ch Ste-Foy, QC G1V 0A6, Canada

**Keywords:** freshwater, bacteria, protist, lotic, *Cyanobacteria*

## Abstract

Streams and rivers convey freshwater from lands to the oceans, transporting various organic particles, minerals, and living organisms. Microbial communities are key components of freshwater food webs and take up, utilize, and transform this material. However, there are still important gaps in our understanding of the dynamic of these organisms along the river channels. Using high-throughput 16S and 18S rRNA gene sequencing and quantitative PCR on a 11-km long transect of the Saint-Charles River (Quebec, CA), starting from its main source, the Saint-Charles Lake, we show that bacterial and protist community structures in the river drifted quickly but progressively downstream of its source. The dominant Operational Taxonomic Units (OTUs) of the lake, notably related to *Cyanobacteria*, decreased in proportions, whereas relative proportions of other OTUs, such as a *Pseudarcicella* OTU, increased along the river course, becoming quickly predominant in the river system. Both prokaryotic and protist communities changed along the river transect, suggesting a strong impact of the shift from a stratified lake ecosystem to a continuously mixed river environment. This might reflect the cumulative effects of the increasing water turbulence, fluctuations of physicochemical conditions, differential predation pressure in the river, especially in the lake outlet by benthic filter feeders, or the relocation of microorganisms, through flocculation, sedimentation, resuspension, or inoculation from the watershed. Our study reveals that the transit of water in a river system can greatly impact both bacterial and micro-eukaryotic community composition, even over a short distance, and, potentially, the transformation of materials in the water column.

## 1. Introduction

Microorganisms are of fundamental importance for the functioning of freshwater ecosystems and aquatic biogeochemical cycles. With autotrophic, heterotrophic, or mixotrophic metabolisms, the freshwater microbial communities transform and recycle particulate and dissolved compounds available in water [1,2,3,4,5]. These microorganisms are also the basis of aquatic food webs sustaining, for example, larger eukaryotic life forms [4,5,6,7]. According to numerous taxonomic surveys, *Betaproteobacteria*, *Actinobacteria*, and *Bacteroidetes* are the major bacterial lineages detected in freshwater [5,8,9,10], while alveolates, cryptophytes, and stramenopiles are the main eukaryotic lineages [11,12,13,14].

Nonetheless, freshwater microbial communities have been mainly investigated in lake ecosystems, while their counterparts in other freshwater ecosystems such as rivers or streams remain poorly explored. Consequently, it is not clear to what extent microbial communities differ between these two kinds of ecosystems [5,9,15]. However, significant differences could be expected since lotic ecosystems, characterized by running waters, contrast in many ways from lentic ecosystems with standing waters. Indeed, whereas lentic ecosystems are rather steady habitats with long water residence time, the continuous flow of water in lotic ecosystems moves irremediably materials and microorganisms downstream. For this reason, rivers could be mistakenly regarded as simple passive pipes in which water, elements, and microbial communities are just passively transported by the river. However, numerous studies have now demonstrated that various transformations occur in running waters resulting in a totally different water in terms of elements and microbial community composition between the source and the mouth of a river [16,17,18,19]. Furthermore, it has been shown that microbial communities are largely involved in these transformations [2,19,20,21,22].

The River Continuum Concept described in 1980 by Vannote and colleagues proposed that biological communities in rivers change progressively downstream from the headwater to the river mouth, using nutrients transformed by upstream communities [23]. Previous studies have explored the dynamics of microbial communities in large rivers, such as the Saint-Lawrence River in Canada [24], the Changjiang River in China [25], the Danube River in Europe [15,26], the River Thames in the UK [27], or the River Spree in Germany [28], from their headwater to their river mouth. These studies analyzed the microbial communities over hundreds or thousands of kilometers of rivers with sampling points separated by tens to hundreds of kilometers. They all observed progressive changes of the microbial communities and an overall decline of phytoplankton communities along the river path, supporting the River Continuum Concept in large fluvial systems.

However, river ecosystems are not limited to large fluvial systems and can be highly diverse in terms of width, slope, flow rate, path, and source type (lake or groundwater). Rivers can also be subject to more or less anthropogenic pressures, going through urbanized, agricultural, or forest areas, or been transformed through artificialization of the river banks and modifications of the watercourse for hydropower generation [29], with potential consequences on the microbial communities and processes. Considering the recognized importance of river microbial communities in local and global biogeochemical cycles, bioremediation, and food webs, finer scale investigations are needed to better understand these freshwater microbial populations, which could react and respond differently from lake microbial communities to environmental modifications such as climate change or water pollution.

The aim of this study was to investigate fluvial microbial community composition and short-scale dynamics within the first 11 km of a meandering river originating from a lake. We examined how changes in environmental conditions, as well as inputs from confluences, impacted the bacterial and protist community composition in the Saint-Charles River, Quebec (Canada). Combining rRNA gene high-throughput sequencing and quantitative Polymerase Chain Reaction (PCR) with physical and chemical parameters at 25 sampling points, we aimed to better understand the fine changes in the microbial community structures along the river path. In studying the transition between a stratified lake to a running habitat, we also looked for indicator lineages that could be specific of lotic or lentic environments to estimate to what extent riverine and lake microbial communities differ.

## 2. Materials and Methods

### 2.1. Study Area

**Figure 1:**
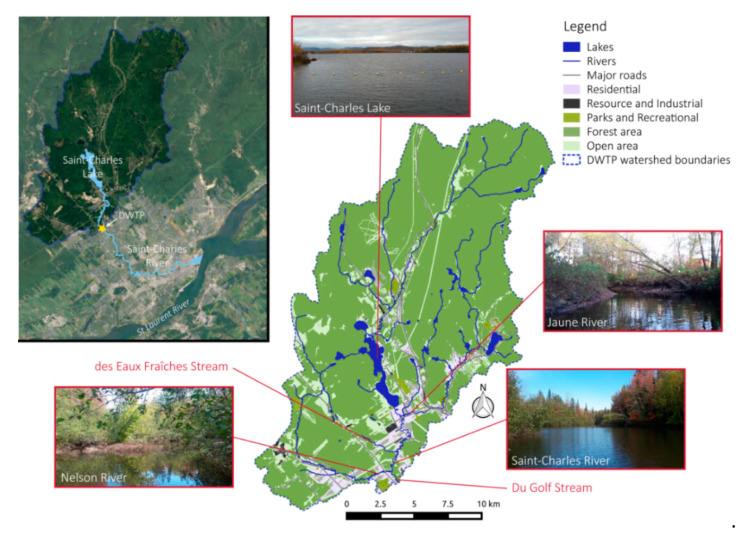
Map of the studied area. Map of the Saint-Charles River showing the location and the visual appearance of the principal tributaries (the Saint-Charles Lake, the Jaune River, and the Nelson River). At the center, the map represents the land occupancy of the watershed before the Drinking Water Treatment Plant (DWTP), showing the most downstream sampling points of this study.

The Saint-Charles River is one of the main drinking water sources of Quebec City (Canada). This river originates from the Saint-Charles Lake, traveling through Quebec City and flowing into the Saint-Lawrence River (Figure 1). The river flow is mainly controlled using flood gates associated with a dam located at the Saint-Charles Lake outlet. Our study focused on the upper part of the river located between the Saint-Charles Lake and the raw water intake of the Loretteville Drinking Water Treatment Plant (DWTP, Quebec City, Canada), located along the Saint-Charles River, 11-km downstream of the Saint-Charles Lake (Figure 1). At the sampling date, the Saint-Charles River exhibited a typical end of summer condition with an estimated flow rate of 1 m^3^.s^−1^ (Ministère de l’Environnement et de la Lutte contre les Changements Climatiques, MELCC), and the waters took approximately 3 h to flow from the dam to the DWTP (L. Collin, pers. com.). This part of the Saint-Charles River draws numerous meanders with various wetlands [30] and is of order 5, with an average slope of 2%, silt and sand bottom [31], and 22 m in width on average. The Saint-Charles River is evenly fed by the Saint-Charles Lake and two main tributaries (the Jaune River and the Nelson River) with two other minor streams (the des Eaux Fraîches Stream and the du Golf Stream) (Figure 1). The water intake watershed has a surface area of 348 km^2^ and is primarily characterized by forest and residential areas and includes several mining areas (granite, gravel, and sand) [32] (Figure 1). The Saint-Charles Lake, which is used as a drinking water reservoir, covers an area of 3.6 km^2^ with a maximal depth of 16.5 m and is at an advanced mesotrophic stage [30]. The Jaune River is an order 4 river, flowing into the Saint-Charles River approximately 1 km downstream of the outlet of the Saint-Charles Lake (Figure 1). The sub-basin of the Jaune River is primarily characterized by forest and low urban development density [30]. The des Eaux Fraîches Stream, order 3, flows into the Saint-Charles River approximately 4 km upstream of the water intake (Figure 1). Its watershed includes agricultural areas (poultry farm). The Nelson River, order 4, flows into the Saint-Charles River approximately 1 km upstream of the water intake (Figure 1). The sub-basin of the Nelson River is mainly characterized by forest with some agricultural areas (poultry farm and cereal crop) and military installations [30]. Finally, the du Golf Stream is a very small stream that goes through a golf course and flows into the Saint-Charles River a few meters upstream of the water intake (Figure 1).

### 2.2. Sampling Sites and Methods

Water sampling was carried out on 3 October 2017 at 20 different sites in the Saint-Charles River, at one site in each tributary, as well as in one site in the Saint-Charles Lake for a total of 25 sampling sites. At each site, surface water samples (15 cm below the surface) were collected for microbiological and physicochemical analyses. For microbiological analyses, 150 mL of surface water (a combination of 50 mL collected at the center of the River, 50 mL near the left bank, and 50 mL near the right bank) were collected per site using sterile syringes and filtered on site directly through a 0.22 µm Sterivex^TM^ unit (MF-Millipore; Millipore Corporation, Burlington, MA, USA) without prefiltration. This sampling process was carried out in duplicate for a total of 50 samples. Filters were stored at 4 °C during the sampling day and then frozen at −80 °C in the laboratory until further analyses. Physicochemical parameters were measured on 1 L of surface water (a combination of 340 mL at the center of the river, 340 mL near the left bank, and 340 mL near the right bank). Water temperature was measured in situ at each sampling location.

### 2.3. DNA Extraction, DNA Amplification, and Sequencing

Before DNA extraction, 0.2 µm filters were removed from their casing and cut into small pieces with a sterile scalpel as described in Cruaud et al. (2017) [33]. DNA extraction was subsequently performed using the AllPrep DNA/RNA mini kit (QIAGEN, Hilden, Germany), as described in Cruaud et al. (2017) [33]. Bacterial 16S rRNA genes and eukaryotic 18S rRNA genes were amplified and sequenced following a two-step PCR library preparation, as detailed in Cruaud et al. (2017) [34]. During the first PCR step, the V3–V4 regions of the bacterial 16S rRNA genes (rDNA) and the V4 region of the eukaryotic 18S rDNA were amplified using primers Bakt341F and Bakt805R [35] and primers E572F and E1009R [36], respectively. PCR conditions were the same as in Cruaud et al. (2019) [10] for bacteria (hybridization temperature of 58 °C) and in Cruaud et al. (2019) [14] for eukaryotes (hybridization temperature of 55 °C). Illumina MiSeq adaptors and barcodes were subsequently added during the second PCR step; then, PCR products were pooled, purified, and paired-end sequenced on an Illumina MiSeq sequencer using a V3 MiSeq sequencing kit (2 × 300 bp) at the Institut de Biologie Intégrative et des Systèmes (IBIS) sequencing platform (Université Laval, Canada). The raw sequencing data have been submitted to the NCBI database under BioProject accession number PRJNA541322.

### 2.4. Sequencing Analyses

Sequence quality controls were performed on the raw sequence dataset with FastQC v0.11.5 [37]. Paired-end reads were merged using FLASH v2.2.00 [38] with default parameters and extended maximum overlap length (300). Afterward, CUTADAPT v1.12 [39] was used to sort paired reads by gene region, remove primers, and filter out sequences shorter than 350 bp. Then, sorted sequences were dereplicated and clustered into Operational Taxonomic Units (OTUs, 97% similarity); then, putative chimeric sequences and singletons were removed using VSEARCH v.2.3.4 [40]. Finally, taxonomic assignment of the sequences was performed using the *Mothur* Bayesian classifier [41] on the SILVA database (release 132; April 2018, https://www.arb-silva.de/) for bacteria and on the Protist Ribosomal Reference database (PR2, Version 4.11.1, December 2018, [42] for eukaryotes. Analysis scripts and documentation are available in a GitHub repository (https://github.com/CruaudPe/MiSeq_Multigenique). Then, for bacteria, OTUs affiliated with *Eukaryota*, *Archaea*, or *Chloroplast* were removed and results were rarefied using the *sub.sample* command of the *Mothur* pipeline to have the same number of sequences per sample (36,180 sequences per sample, corresponding to the lowest number of sequences in one sample). For eukaryotes, OTUs affiliated with *Bacteria*, *Archaea*, or *Metazoa* were removed, and results were rarefied to 10,075 sequences per sample, corresponding to the lowest number of sequences in one sample.

### 2.5. Quantitative Real-Time PCR

The bacterial abundance in each sample was estimated by quantitative PCR (qPCR) targeting the bacterial 16S rRNA gene. Amplifications were performed in triplicates with a 7500 Fast Real-Time system (Applied Biosystems) in a final volume of 25 µL using Brilliant III UltraFast QPCR Master Mix (Agilent Technologies, Santa Clara, CA, USA), 1 ng of crude DNA template, and 700 nmol.L^−1^ of 16S rRNA bacterial primers (BACT1369F and PROK1492R [43], annealing temperature of 58°C). Standard curves were obtained in triplicate with a serial dilution (10^2^–10^6^ copies per reaction) of genomic DNA from *Polynucleobacter asymbioticus* (DSM18221). The efficiency of the qPCR was close to 99%, and the R^2^ of standard curves were close to 0.996. qPCR results were expressed in 16S rRNA gene numbers per milliliter of filtered water.

### 2.6. Physicochemical Parameters

Standard physicochemical water quality parameters (UV absorbance at 254 nm, alkalinity, total nitrogen, total organic carbon (TOC), conductivity, apparent color, NO_3_^−^/NO_2_^−^, pH, total phosphorus, and turbidity) were measured in the certified water quality laboratory of Quebec City following standard procedures [44].

### 2.7. Statistical Analyses

All statistical analyses (Bray–Curtis indexes, Unweighted Pair Group Method with Arithmetic mean (UPGMA) and Pearson’s correlation tables) were conducted using the software environment R (v.3.4.4) and the RStudio toolkit (v.1.0.143) implemented with *Vegan* [45], *dendextend* [46], and *gplot* [47] packages. In order to highlight some interesting patterns, two different subsets of our dataset were generated to retain only the most dominant OTUs: (i) the “subset 0.1%”, which retains only the OTUs with a number of sequences of at least 0.1% of the total number of bacterial or eukaryotic sequences as appropriate, and (ii) the “subset 0.01%” containing only the OTUs with a number of sequences of at least 0.01% of the total number of bacterial or eukaryotic sequences as appropriate.

Correlations among and between the dominant bacterial OTUs, dominant eukaryotic OTUs (subset 0.1% of the total number of bacterial and eukaryotic sequences, relative proportions along the river), and the environmental parameters were investigated using Pearson’s correlation analyses. Results were represented as a network using the software environment R implemented with the *igraph* package [48]. Bacterial OTUs, eukaryotic OTUs, and environmental parameters were defined as nodes and position of the nodes were determined using a force-directed layout calculated with the Pearson’s correlations (Fruchterman–Reingold layout algorithm, using the weight parameter defined with Pearson’s correlation coefficient to increase the attraction/repulsion forces among nodes connected by higher coefficients). Connecting links (edges) represented only the Pearson scores with a *p*-value < 0.01 for readability.

To better understand the worldwide distribution of OTUs of interest, we also conducted a similarity search with the Integrated Microbial Next Generation Sequencing (IMNGS, https://www.imngs.org, last accessed 19.05.2019; [49]) server. IMNGS is an online platform that allows users to conduct comprehensive searches of Small Subunit (SSU) rRNA gene sequences against prokaryotic 16S rRNA gene amplicon datasets available in SRA (Sequence Read Archive) retrieved from the International Nucleotide Sequence Database [49].

## 3. Results

### 3.1. Environmental Parameters

**Figure 2:**
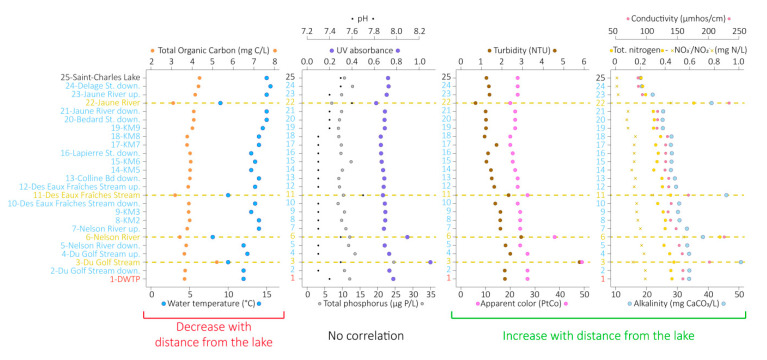
Variations of the environmental and physicochemical parameters measured at each sampling point. Environmental parameters are clustered according to their correlation with the distance from the lake. Down, Downstream; Up, Upstream.

Various physicochemical parameters were measured for the 25 water samples. Total organic carbon (TOC) and water temperature decreased progressively with distance from the lake (R^2^ = −0.85 and −0.84, respectively, Figure 2). TOC decreased from 4.36 mg of carbon per liter (mg C.L^−1^) in the lake to 3.63 mg C.L^−1^ at the DWTP intake and were lower in the tributaries (3.2 mg C. L^−1^ in average), except for the du Golf Stream (5.2 mg C.L^−1^). Water temperatures decreased from 15 °C in the Saint-Charles Lake to 12 °C at the DWTP intake and were lower in the tributaries (9.2 °C in average). 

By contrast, apparent water color, turbidity, total nitrogen, nitrates and nitrites (NO_3_^−^/NO_2_^−^), conductivity, and alkalinity increased gradually with the distance from the lake (R^2^ = 0.72, 0.88, 0.9, 0.91, 0.91, and 0.94, respectively, Figure 2). Apparent water color increased from 23 Platinum–Cobalt (PtCo) units in the Saint-Charles Lake to 27 PtCo units at the DWTP and was higher in the tributaries (38 PtCo units in average), except in the Jaune River (20 PtCo units). Similarly, turbidity varied from 1.24 Nephelometric Turbidity Units (NTU) in the lake to 2.14 NTU at the DWTP, and higher values were measured in the tributaries (3.7 NTU in average), except in the Jaune River (0.71 NTU). Total nitrogen varied from 0.206 mg of nitrogen per liter (mg N.L^−1^) in the lake to 0.456 mg N.L^−1^ at the DWTP and was higher in the tributaries (0.6 mg N.L^−1^ in average). NO_3_^−^/NO_2_^−^ increased from 0.01 mg N.L^−1^ in the Saint-Charles Lake to 0.24 mg N.L^−1^ at the DWTP and were higher in the tributaries (0.43 mg N.L^−1^ in average), except in the du Golf Stream (0.14 mg N.L^−1^). Conductivity varied from 86.2 µmhos.cm^−1^ in the Saint-Charles Lake to 159 µmhos.cm^−1^ at the DWTP and was higher in the tributaries (207.5 µmhos.cm^−1^ in average). Similarly, alkalinity varied from 18.1 mg.L^−1^ CaCO_3_ in the lake to 33.8 mg.L^−1^ CaCO_3_ at the DWTP and were higher in the tributaries (43.9 mg.L^−1^ CaCO_3_ in average).

UV absorbance, total phosphorus, and pH did not correlate with the distance from the lake (R^2^ = 0.35, 0.33, and −0.61, respectively, Figure 2). UV absorbance varied from 0.66 to 0.77 in the Saint-Charles River, was slightly lower for the Jaune River (0.62), and was higher for the Nelson River and the du Golf Stream (0.89 and 1.1, respectively). Total phosphorus varied from 8.8 to 13.6 µg of phosphorus per liter (µg P.L^−1^) in the Saint-Charles River. The lowest concentration was measured in the Jaune River (7 µg P.L^−1^), the highest concentration was measured in the du Golf Stream (24.6 µg P.L^−1^), while the total phosphorus concentrations were quite similar to the Saint-Charles River for the other tributaries. Finally, pH varied from 7.3 to 7.5 in the Saint-Charles River and was higher in the tributaries (7.6 in average).

### 3.2. Microbial Community Structures

The bacterial and eukaryotic diversity was analyzed by 16S and 18S rDNA amplicon sequencing (25 × 2 duplicates). A total of 1,809,000 quality-filtered reads were obtained for bacteria and 503,750 quality-filtered reads were obtained for eukaryotes. Since bacterial and eukaryotic communities from duplicate samples strongly clustered together in UPGMA dendrogram analyses, an average relative proportion of each OTU was calculated for each sampling location for the subsequent analyses. Moreover, only OTUs detected in both duplicates for each sample were selected for all subsequent analyses, resulting in a total of 5476 bacterial OTUs and 1525 eukaryotic OTUs (97% threshold). 

**Figure 3:**
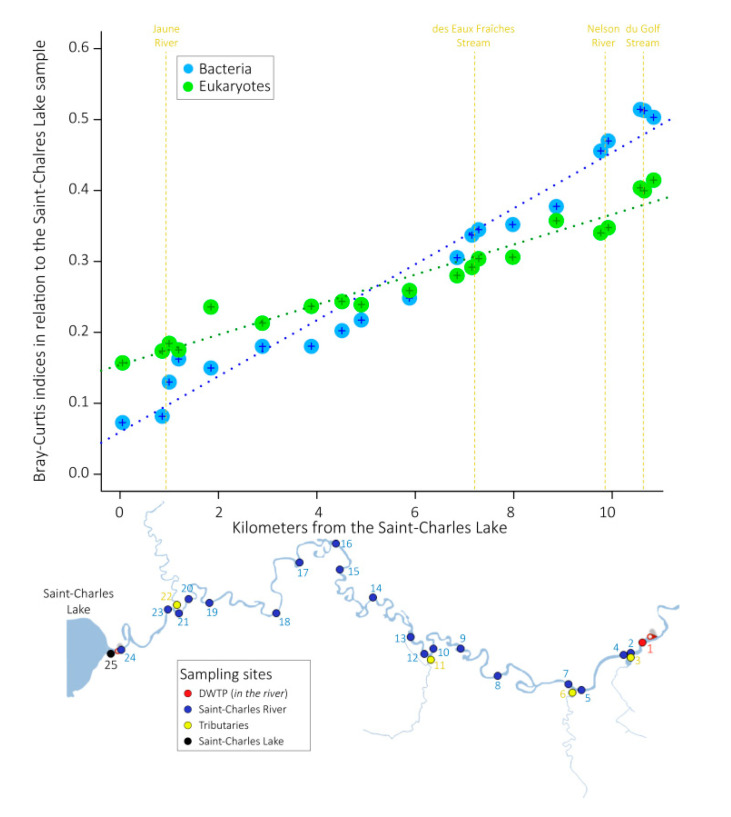
Evolution of the Bray–Curtis dissimilarity indices calculated between samples collected from the lake and samples collected along the water course of the Saint-Charles River. Blue and green dotted lines represent linear regressions between the distance from the lake and the Bray–Curtis dissimilarity indices for bacterial and eukaryotic datasets, respectively. Locations of the tributaries are illustrated by the yellow dotted lines.

Microbial community structures were compared using Bray–Curtis dissimilarity indices (ranging from 0, the two samples have the same microbial community composition, to 1, the two samples do not share any species). We first compared the Saint-Charles Lake sample with samples collected in the Saint-Charles River. For both bacterial and eukaryotic communities, Bray–Curtis indices increased progressively downstream of the lake and were strongly correlated with the distance from the lake (measured as the cumulative water channel distance upstream, R^2^ = 0.96 and 0.95 for the bacterial communities and eukaryotic communities, respectively, Figure 3). For bacterial communities, Bray–Curtis indices increased from 0.07 between the lake and the first sampling site (48 m downstream) to 0.5 between the lake and the DWTP, 11 km downstream. For eukaryotic communities, Bray–Curtis indices increased from 0.16 between the lake and the first sampling site to 0.41 between the lake and the DWTP (Figure 3).

**Figure 4:**
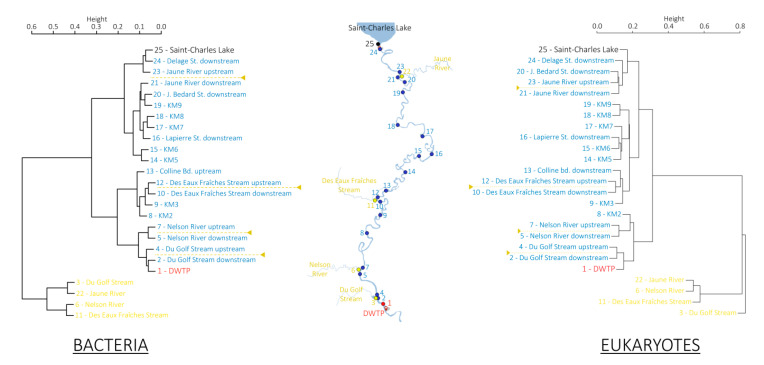
Unweighted Pair Group Method with Arithmetic mean (UPGMA) dendrograms based on Bray–Curtis distances representing bacterial communities (left) and eukaryotic communities (right). Samples collected from the Saint-Charles River are represented in blue and red (the most downstream sampling point), those collected from the tributaries in yellow and sample collected from the Saint-Charles Lake in black. Locations of confluences between the Saint-Charles River and the tributaries are illustrated by the yellow dotted lines on the UPGMA dendrograms.

Then, Bray–Curtis indices were calculated between all sample pairs, including the four tributaries, and visualized on UPGMA dendrograms (Figure 4). For both bacterial and eukaryotic communities, samples from the Saint-Charles Lake and the Saint-Charles River clustered together, while samples from the tributaries formed a separate group on the dendrograms. Considering the Saint-Charles River sampling sites and tributaries, Bray–Curtis indices were 0.64 and 0.81 on average for bacterial and eukaryotic communities, respectively, while the indices were 0.23 and 0.24 on average considering only the Saint-Charles River sampling sites (Figure 4). 

### 3.3. Presence/Absence of OTUs

The number of bacterial OTUs ranged from 816 to 1020 OTUs per sample (average of 987 OTUs per sample) and did not correlate with the distance from the lake (R^2^ = −0.09). On contrary, the number of eukaryotic OTUs increased with distance from the lake (R^2^ = 0.73, *p*-value < 0.001) ranging from 303 to 413 OTUs (average of 363 OTUs per sample).

Considering all the detected OTUs in the Saint-Charles River, including rare and dominant OTUs (“No Subset” in Figure S1), the large majority of OTUs were intermittently detected between the lake and the DWTP intake (“unstable OTUs”, 88% and 82.6% for the bacterial and eukaryotic OTUs, respectively, gray in Figure S1), which was likely due to the differential probability of detection of rare OTUs using a high-throughput sequencing method. Some OTUs were detected in all samples from the lake to the DWTP (“stable OTUs”, 5.9% and 10.8% for the bacterial and eukaryotic OTUs, respectively, blue in Figure S1), while emerging OTUs (OTUs not detected in the lake but detected in the Saint-Charles River and in all the samples downstream) and lost OTUs (OTUs detected in the lake and in the first sample(s) of the Saint-Charles River then not detected anymore downstream) represented the smallest proportions (5.7% and 6.6% for the bacterial and eukaryotic OTUs, respectively, red and green in Figure S1).

**Figure 5:**
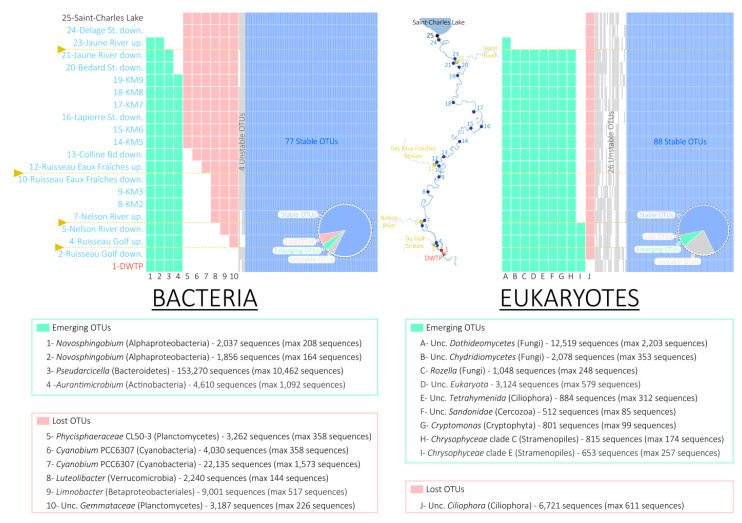
Detection pattern of the dominant bacterial and eukaryotic Operational Taxonomic Units (OTUs) along the water course of the Saint-Charles River (presence/absence, subset 0.1% of the total number of bacterial and eukaryotic sequences). Colored cells (green, red, gray, or blue) illustrate the presence of the OTU, while the white cells illustrate the absence of the OTU in the river. The tributary locations are represented by the yellow lines. Numbers indicated after the OTU names are the total number of sequences corresponding to this OTU in the entire dataset and the maximum number of sequencing of this OTU detected in a sample (in brackets). Proportions of stable (blue), unstable (gray), emerging (green), and lost (red) OTUs are represented by the pie charts.

In contrast, when only the dominant OTUs were considered (OTUs with relative proportions higher than 0.01% or 0.1% of the total number of bacterial and eukaryotic sequences), the proportion of stable OTUs increased and reached 84.6% and 71% of the bacterial and eukaryotic OTUs, respectively, for the 0.1% subset. Only a few OTUs were lost (six bacterial OTUs and one eukaryotic OTU for the 0.1% subset) or emerged (four bacterial OTUs and nine eukaryotic OTUs for the 0.1% subset, Figure 5 and Figure S1).

The six bacterial OTUs lost disappeared successively from 6.8 km downstream of the lake (between the 13th and 14th sampling points, Figure 5). Among them, the major OTU, affiliated with *Cyanobium* PCC6307 (*Cyanobacteria*), was represented by a total of 22,135 sequences in our dataset (maximum of 1573 sequences in the lake, OTU 7 in Figure 5). The results of querying the IMNGS 16S rRNA database indicated that this OTU sequence has been frequently detected in lakes all around the world (Figure S2). The single eukaryotic OTU lost disappeared between the first and the second sampling points and was affiliated with Unc. *Ciliophora* (*Alveolata*, total of 6721 sequences in our dataset, OTU J in Figure 5). 

The four emerging bacterial OTUs appeared between the 24th sampling point (48 m downstream of the lake) and the 19th sampling point (1.8 km downstream of the lake, Figure 5). Among them, the major OTU, affiliated with *Pseudarcicella* (*Bacteroidetes*), was represented by a total of 153,270 sequences (maximum 10,462 sequences in the Jaune River, OTU 3 in Figure 5). The results of querying the IMNGS 16S rRNA database indicated that this OTU sequence has been mainly detected in rivers or streams all around the world (Figure S2). The nine emerging eukaryotic OTUs mainly appeared between the 24th and the 21th sampling points (located 48 m and 1 km downstream of the lake, respectively) with one OTU emerging between the 7th and the 5th sampling points (located 9.8 and 9.9 km downstream of the lake, at the junction of the Nelson River with the Saint-Charles River, Figure 5). Among them, the main OTU was affiliated with Unc. *Dothideomycetes* and was represented by a total of 12,519 sequences in our dataset with a maximum of 2203 sequences in the Jaune River (OTU A in Figure 5).

Potential origins of bacterial and eukaryotic OTUs detected at the DWTP sampling site were also investigated (“DWTP: Origins of OTUs” in Figure S1). Considering all OTUs detected at the DWTP sampling site including rare and dominant OTUs (“No subset” in Figure S1, “DWTP OTUs”), most of the OTUs were also identified both in the lake and in tributaries or only in tributaries and not in the lake (green and yellow in Figure S1). Smaller proportions of the DWTP OTUs were not detected in the lake nor in the tributaries (red in Figure S1) or only in the lake but in no other tributaries (black in Figure S1). Proportions of DWTP OTUs that were also detected both in the lake and in the tributaries increased when considering only the main OTUs (subsets 0.01% and 0.1% in Figure S1), reaching 84.5% and 64.9% of the bacterial and the eukaryotic OTUs, respectively, for the 0.1% subset (green in Figure S1).

### 3.4. Correlations between Bacterial OTUs, Eukaryotic OTUs, and Environmental Parameters

**Figure 6:**
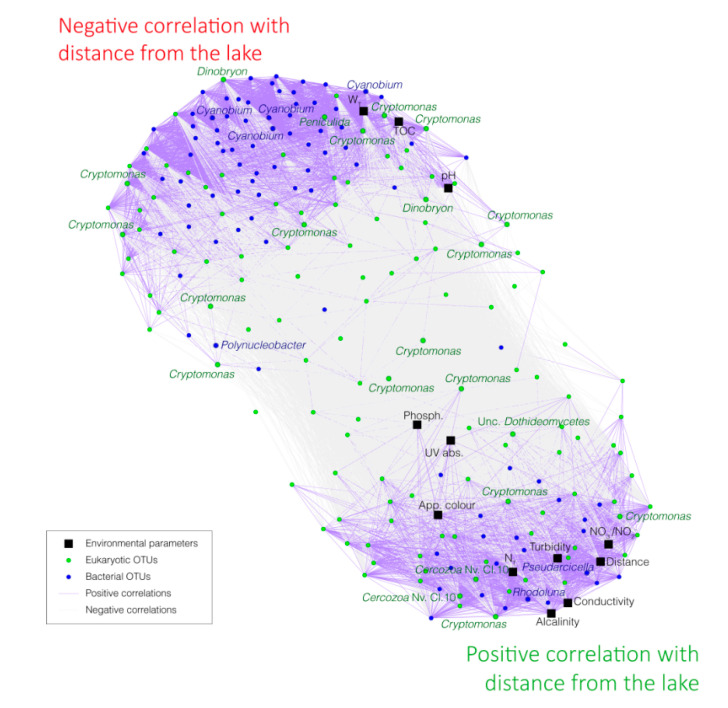
Co-varying network representing correlations among and between the dominant bacterial OTUs, dominant eukaryotic OTUs (subset 0.1% of the total number of bacterial and eukaryotic sequences), and the environmental parameters. A network was constructed based on the significant Pearson’s correlation coefficients (*p*-value < 0.01). Nodes represent bacterial OTUs (blue), eukaryotic OTUs (green), and environmental parameters (black square). Solid links (edges) represent a correlation between bacterial and eukaryotic OTUs as well as environmental parameters. Purple links and gray links represent positive and negative correlations, respectively.

Correlations among and between the dominant bacterial and eukaryotic OTUs (subset 0.1%, blue and green dots in Figure 6) as well as with the environmental parameters (black square in Figure 6) were carried out. Two main clusters of bacterial and eukaryotic OTUs associated with different environmental parameters were detected: (i) OTUs and environmental parameters decreasing in relative proportions or values with distance from the lake (62 bacterial OTUs and 41 eukaryotic OTUs with significant negative correlation with distance from the lake, *p*-value < 0.05, e.g., OTUs affiliated with *Cyanobium* PCC6307, *Dinobryon, Cryptomonas, Peniculida*, water temperature and TOC, Figure 6) and (ii) OTUs and environmental parameters increasing in relative proportions or values with distance from the lake (23 bacterial OTUs and 44 eukaryotic OTUs with significant positive correlation with distance from the lake, *p*-value < 0.05, e.g., OTUs affiliated with *Pseudarcicella*, *Rhodoluna*, other *Cryptomonas* OTUs, *Cercozoa* Novel Clade 10, turbidity and total nitrogen concentrations, Figure 6). Finally, other OTUs and environmental parameters did not show any significant correlation with the distance from the lake (six bacterial OTUs and 40 eukaryotic OTUs, *p*-value < 0.05, e.g., OTUs affiliated with *Polynucleobacter*, other OTUs of *Cryptomonas*, phosphorus concentrations, as shown in Figure 6).

### 3.5. Variations in the Relative Proportions of the Main Bacterial Taxa

**Figure 7:**
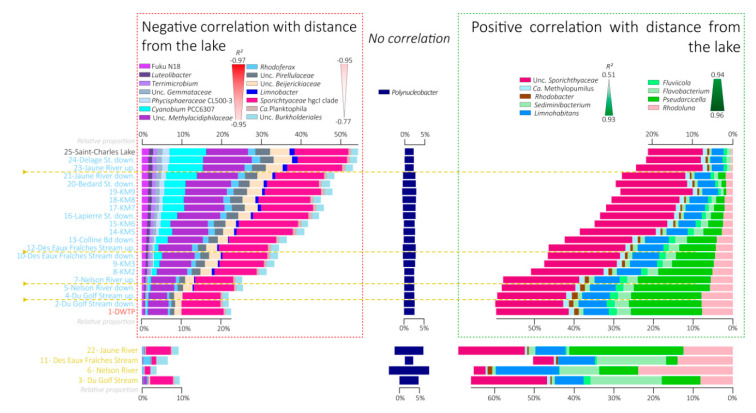
Bar chart representing the variation in relative proportions of the main bacterial lineages. Variations in the Saint-Charles River from the lake to the DWTP are represented at the top, and variations in the tributaries are represented at the bottom. Bacterial lineages are sorted in bar charts by correlation with distance from the lake (R^2^ scale in the caption) with the lineages the more negatively correlated with distance from the lake at the left and lineages that are more positively correlated with distance from the lake at the right. The three categories are defined based on statistically significant correlation (negative correlation, no correlation, and positive correlation, *p*-value < 0.05). The other bacterial lineages with low proportions (<0.5% of total sequences in the entire dataset, or a minimal number of sequences of 9045) are not represented on this figure (representing between 13.7% and 21.8% of sequences). Unc.: Unclassified.

Most of the bacterial sequences were affiliated with *Actinobacteria* (35.8% on average). The *Actinobacteria* phylum did not show a clear pattern according to the distance from the lake with the *Sporichthyaceae* hgcI clade and *Candidatus* Planktophila slightly decreased with distance from the lake (R^2^ = −0.77 and −0.67, respectively), while *Candidatus* Rhodoluna showed the opposite trend (R^2^ = 0.96) (Figure 6 and Figure 7).

*Bacteroidetes* were the second most abundant bacterial phylum, representing 17.6% sequences on average. This phylum, as well as the lineages belonging to it, increased in relative proportions with distance from the lake (Figure 7 and Figure S3). Relative proportions of sequences affiliated with *Pseudarcicella*, *Flavobacterium*, *Fluviicola*, and *Sediminibacterium* were strongly correlated with distance from the lake (R^2^ = 0.95, 0.94, 0.94, and 0.91, respectively, Figure 6, Figure 7 and Figure S3).

*Betaproteobacteria* were represented by 15.8% of bacterial sequences on average. Some lineages belonging to this phylum decreased with distance from the lake in relative proportions, such as *Limnobacter* (R^2^ = −0.87), while others increased, such as *Limnohabitans* (R^2^ = 0.93), and others did not correlate with distance from the lake, such as *Polynucleobacter* (R^2^ = −0.31) (Figure 6 and Figure 7).

Relative proportions of sequences affiliated with *Verrucomicrobia* phylum (11.7% on average of total sequences, purple in Figure 7), such as Fuku N18 (R^2^ = −0.97), *Luteolibacter* (R^2^ = −0.97), or Unc. *Methylacidiphilaceae* (R^2^ = −0.95), as well as *Planctomycetes* phylum (4% on average of total sequences, slate gray in Figure 7), and *Cyanobacteria* (3.2% on average of total sequences, turquoise in Figure 7), decreased with distance from the lake (Figure 6, Figure 7 and Figure S3).

### 3.6. Variations in the Relative Proportions of the Main Eukaryotic Taxa

**Figure 8:**
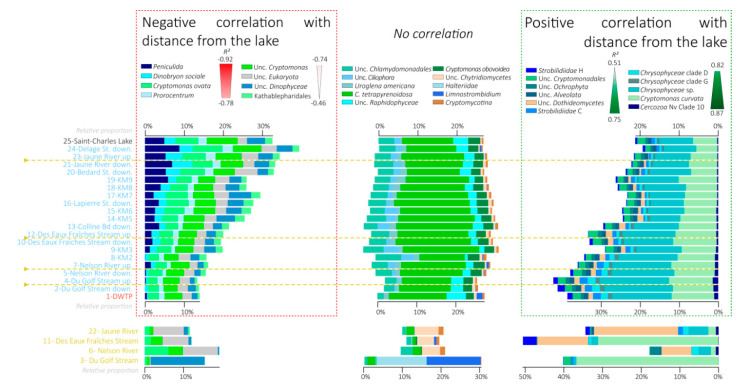
Bar chart representing the variation in relative proportions of the main eukaryotic lineages. Variations in the Saint-Charles River from the lake to the DWTP are represented at the top and variations in the tributaries are represented at the bottom. Eukaryotic lineages are sorted in bar charts by correlation with distance from the lake (R^2^ scale in the caption) with the lineages the more negatively correlated with distance from the lake at the left and lineages the more positively correlated with distance from the lake at the right. The three categories are defined based on statistically significant correlation (negative correlation, no correlation, and positive correlation, *p*-value < 0.05). The other eukaryotic lineages with low proportions (<0.5% of total sequences in the entire dataset or a minimal number of sequences of 2519) are not represented on this figure (representing between 14.6% and 21.5% of sequences). Unc.: Unclassified.

Most of the eukaryotic sequences were affiliated with *Hacrobia* (40% on average, green), mainly within the *Cryptophyceae* class (37.9% on average of total sequences). Various lineages and OTUs affiliated with *Cryptomonas* increased, decreased, or did not correlate with distance from the lake (Figure 6 and Figure 8).

Stramenopiles were the second most abundant eukaryotic phylum with 24.1% on average of total sequences (turquoise in Figure 8). In the same way as *Hacrobia*, no clear pattern of variation in relative proportions were observed for this phylum, with a decrease for some lineages, such as *Dinobyron* (R^2^ = −0.90), or an increase for other lineages, such as various clades of *Chrysophyceae* (R^2^ = 0.82, 0.84, and 0.84 for clade D, clade G, and unclassified *Chrysophyceae*, respectively; Figure 6 and Figure 8).

*Alveolata* represented on average 18.7% of total sequences (blue in Figure 8) and were mainly affiliated with *Ciliophora* (11.7% on average of total sequences). No clear pattern was observed regarding the distance from the lake except for the *Peniculida* lineage, which decreased in relative proportions with distance from the lake (R^2^ = −0.92) (Figure 6 and Figure 8).

*Fungi* represented on average 5.6% of total sequences (brown in Figure 8) and were detected in higher relative proportions in the tributaries, with the *Dothideomycetes* lineage increasing slightly in relative proportions with distance from the lake (R^2^ = 0.7) (Figure 6 and Figure 8).

### 3.7. Quantitative Real-Time PCR

The relative abundances of *Bacteria* were estimated by qPCR (Figure S4). Bacterial relative abundances fluctuated between 9.35 ± 0.44 × 10^5^ and 3.25 ± 0.59 × 10^5^ 16S rRNA gene per mL of filtered water in the Saint-Charles River. The bacterial relative abundance in the Saint-Charles Lake and at the DWTP intake were of the same order of magnitude (4.94 ± 0.98 × 10^5^ and 6.40 ± 0.93 × 10^5^ 16S rRNA gene copies per mL of filtered water, respectively), and no clear correlation was detected between the bacterial relative abundances and the distance from the Saint-Charles Lake.

## 4. Discussion

### 4.1. Progressive Changes of the Global Microbial Community along the River Course

In this study, we investigated spatial changes in the microbial community structure in the first 11 km of the Saint-Charles River (Quebec, Canada, Figure 1). We observed a progressive increase of Bray–Curtis indices between bacterial and eukaryotic community structures from the lake (source of the river) and those from the 20 sampling sites downstream in the Saint-Charles River (Figure 3). This reveals a progressive modification of both bacterial and protist community structures within the first kilometers of the river and suggests that the gradual change of microbial community from upstream to downstream observed in large and long rivers [15,25,26,27] also occurs over short distance in smaller rivers.

The environmental parameters and microbial community structures of the Saint-Charles River contrasted from those of its tributaries (Figure 2 and Figure 4), which was probably due to the different watershed and watercourse characteristics (e.g., depth, width, flow rate, land use). However, no particularly marked changes along the gradual modification of the physicochemical characteristics and the microbial community structures were observed at the confluences (Figure 2, Figure 3 and Figure 4), revealing a limited impact of the tributaries on the Saint-Charles River, as previously observed in other rivers [15], which was probably linked to the reduced water flow in the tributaries during the period of sampling (late summer).

Interestingly, the spatial modification of the microbial community structure (maximal Bray–Curtis indices detected between two samples: 0.51 and 0.44 for bacterial and eukaryotic community structure, respectively) was lower than the changes induced by the seasonal variations previously investigated in the Saint-Charles River (maximal Bray–Curtis indices between the summer and winter seasons: 0.92 and 0.98 for the bacterial and eukaryotic community structure, respectively) [10,14]. This result suggests that in contrast to seasonal changes that induce rapid shifts in community composition, spatial changes are associated with a progressive drift of the bacterial and protist community composition, supporting previous observations in other rivers [25,26,50].

### 4.2. Contrasted Dynamics among Microbial Communities

Most of the dominant OTUs were detected in all river samples, and the large majority of the dominant OTUs detected at the end of our 11 km transect were also detected in the lake and in the tributaries feeding the river (Figure 5 and Figure S1). Furthermore, qPCR data indicated that the progressive drift in the microbial community structure was not associated with a loss of abundance (Figure S4). Therefore, these results suggest that the difference in community structure in water at the source of the river and 11 km downstream likely result mainly from changes in relative abundances of the OTUs rather than from the presence of different OTUs.

The correlation analyses between the dominant OTUs indicated two main clusters of OTUs characterized by two opposite patterns of variation along the river course (Figure 6). Almost 80% of the dominant OTUs detected in the samples showed a modification of their relative proportion across the river section with 47.7% of OTUs decreasing with distance from the lake and 31% of OTUs increasing with distance from the lake (Figure 6, Figure 7 and Figure 8). This suggests that the passage from a lentic ecosystem to a lotic ecosystem is associated with a strong modification of the microbial community structuring factors. Interestingly, the ratio of eukaryotic OTUs without a change of proportion along the lake-river section was larger than the proportion of bacterial OTUs (32% vs. 6.6% of the OTUs), suggesting a better resistance of the eukaryotic communities to the changes of ecosystem type than the bacterial communities.

### 4.3. Fall of the Lake Microbial Communities

Among the lineages that seemed to be negatively impacted by the transition between the lake and the river, the *Cyanobacteria* lineage had one of the most pronounced decreases (Figure 5, Figure 7, Figure S2 and Figure S3), supporting previous observations in other lake–river systems [28]. One of the main cyanobacterial OTU affiliated with *Cyanobium* PCC6307 was particularly affected (from 3.7% of the sequence in the lake to below the detection limit after 8 km downstream, OTU 7 in Figure 5 and Figure S3). Sequence comparison with the SRA database indicated that this OTU is mainly detected in lentic ecosystems (similarity search with the IMNGS server, Figure S2), suggesting a better adaptation of this OTU for this kind of environment. Similarly, many other bacterial lineages such as *Verrucomicrobia* (Fuku N18, *Luteolibacter*, *Terrimicrobium*), *Planctomycetes*, some *Betaproteobacteriales* lineages (*Limnobacter*, *Rhodoferax*), as well as eukaryotic lineages affiliated with *Ciliophora*, *Chrysophyceae*, or *Cryptophyceae* decreased in relative proportion with the distance from the lake (Figure 7 and Figure 8). There are multiple hypotheses to explain these falls.

One of the most obvious differences between lakes and rivers is the turbulent flow of water. At the sampling period (end of summer), the Saint-Charles Lake is highly stratified and thus characterized by standing water and steady conditions, whereas the Saint-Charles River is a running water habitat. The water flow in rivers is generally non-uniform and strongly three-dimensional [51]. This feature is even more pronounced in sinuous river with meanders such as the Saint-Charles River [30], which is also known as the River *Kabir Kouba*, meaning the river of the thousands of detours in Huron Wendat first nation language (Figure 1). The water turbulence could expose cells to rapid fluctuations of conditions in terms of light intensity and nutrient concentrations, potentially impacting negatively organisms that lived in the stratified lake, as previously suggested for phytoplankton [28,52].

A decrease in the abundance of lineages could also be explained by the absence of these lineages at the water sampling depth (15 cm below the surface) and could reveal a resettlement of these microorganisms. In response to environmental changes, some microorganisms could form microbial mats, which is an organization often observed in riverine cyanobacterial lineages [53,54,55], and resettle at the river bed or on the surface of macro-algae.

The drift of the microbial community composition might also reflect a modification of the food chain in the river. For instance, lake outlets are often characterized by the presence of numerous invertebrates and abundant filter feeders, as observed at the Saint-Charles Lake outlet (W. F. Vincent, pers. com.). These organisms growing on the large outputs of matter from the lake [56,57,58] might likely contribute to the decline of lake microbial populations by their feeding behavior [58,59,60,61]. The high water turbulence creating by the dam at the lake outlet can also lead to the aggregation of suspended particles in larger aggregates [62,63,64], which could be better retained by the filter feeders [65,66]. The excretion of flocculent organic material, such as extracellular polysaccharides (EPS) by for instance *Cyanobacteria*, might also increase their own flocculation [62,64,67], thus promoting their retention by the filter feeders compared with other lineages, such as *Polynucleobacter* and *Sporichthyaceae*, which seem less impacted by the lake–river transition (Figure 7). In addition, other predators such as zooplankton or protists (Figure 8) might consume the lake-derived microbial communities. Similarly, the occurrence of parasitic lineages, such as chytrids (Figure 8), which are known to be able to terminate phytoplankton species blooms [68], could also contribute to the decrease of these lineages in the river. The decrease or resettlement of microbial organisms could also lead to the decline in proportion of other associated microbial populations. *Verrucomicrobia* lineages (Fuku N18, *Luteolibacter*, *Terrimicrobium*) are among the most affected groups (Figure 7). These lineages are often associated with the degradation of carbohydrates in aquatic ecosystems [69,70]. Cultivated species are involved in the degradation of microbial and algal exopolysaccharides [71], and genes for the assimilation of cyanobacterial and phytoplankton-derived saccharides were identified in genomes of uncultivated lineages, suggesting a close interaction between *Verrucomicrobia* species and the phytoplankton [72]. Therefore, the decline of *Verrucomicrobia* lineages might likely be associated with the *Cyanobacteria* and phytoplankton falls.

Finally, the Saint-Charles River and its tributaries go across watersheds with different characteristics (e.g., agricultural and urbanized areas, military installations, a golf course, car cemeteries), which could bring nutrients but also organic pollutants and toxic substances in the river, leading to spatial fluctuations of water quality. Microorganisms living in the lake might be sensitive to these additional components.

### 4.4. Rise of the Riverine Microbial Communities

While some microbial populations seemed negatively impacted by the river conditions, others increased in relative proportions downstream from the lake (Figure 7 and Figure 8). *Bacteroidetes* lineages were among the main lineages with a gain in relative proportions with the distance from the lake (Figure 7). One of the main dominant OTUs of this lineage, which is affiliated with *Pseudarcicella* (Figure 6, Figure 7 and Figure S3), was a major emerging OTU (OTU 3 in Figure 5). A sequence comparison of this OTU with public databases indicated that this OTU is related to the genus *Aquirula*, which was recently isolated and described from running water systems [73]. Strains of this genus grew chemoorganotrophically and aerobically on pectin and showed an intense red pigmentation putatively due to various carotenoids [73]. Moreover, this OTU has mainly been previously detected in lotic ecosystems (similarity search with the IMNGS server, Figure S2), suggesting a better adaptation of this taxon for turbulent environments.

While most of the OTUs detected in the river were also identified in the lake, four emerging OTUs represented by more than 0.1% of the sequences in the entire dataset were detected in the Saint-Charles River (Figure 5). These emerging OTUs were probably introduced into the river near the most upstream site at which they were first identified. Regarding the *Pseudarcicella*/*Aquirula* OTU, the highest proportion was detected in the Jaune River (28.7%, Figure 7 and Figure S3), and this OTU was detected for the first time just downstream of this tributary (Figure 5, Figure 7 and Figure S3). While the tributaries seemed to have only small overall impacts on the Saint-Charles River physicochemical water quality and community structures (Figure 2, Figure 3, Figure 4, Figure 7 and Figure 8), these results suggest that the tributaries could have an important impact as sources of diversity. As the tributaries, the backwaters and oxbows intercalated into the river continuum as well as a possible terrestrial microbial inoculation could also be potential sources of diversity for the Saint-Charles River. Another hypothesis that might explain the emergence of a novel OTU in the river is the resuspension of fine particles from the water bed. The settling and sedimentation rate increases with the density and diameter of aggregates, meaning that while large particles could settle down in the river, the water turbulence could lead to the resuspension of fine particles from the water bed [74]. This phenomenon could potentially favor the mobilization of free-living microbial populations as well as increase the turbidity, as observed along the river (Figure 2). Interaction with the riverbed could also be a potential driver of change in the river, including macrophytes, biofilms, and potential hyporheic exchange (mixing areas between surface water and groundwater) [75].

Whether the communities were already in the lake water or originate from downstream of the lake, the successful species in the river required being better adapted to the dynamic environment of the river than the lake-adapted populations [76]. This could be the case of the emerging *Pseudarcicella* OTU, which was detected as prevalent in river ecosystems in previous studies (similarity search with the IMNGS server, Figure S2). *Limnohabitans*, with increasing relative proportions with the distance from the lake (Figure 7), has also been proposed to rapidly respond to changes in substrate supply and to be particularly competitive in freshwaters because of their opportunistic lifestyle, with a fast growing rate [5,77,78]. The decrease of some microbial populations better adapted to the lake environment could lead to a decrease in nutrient competition and the liberation of ecological niches. Rapidly growing species that can quickly use available resources (r-strategists) could be favored at the expense of the late summer lake populations with potentially slow growth rates (k-strategists).

The river microbial lineages could also directly benefit from nutrients released by senescent microorganisms of the lake. Several members of *Cytophaga* lineages detected in increasing proportions in the river are known to be cyanobacterial-lysing bacteria and play a role in the decrease of some *Cyanobacteria* population events [79,80,81]. Likewise, parasitic lineages, such as *Fungi*, may grow on phytoplankton blooms [68]. Enzymatic attack, predation, and parasitism by these organisms on the lake microbial populations can also liberate dissolved organic matter and nutrients that would be available for other fast-growing lineages, such as *Limnohabitans* or some *Bacteroidetes* lineages, which are positively correlated with algal-derived dissolved organic carbon (DOC) inputs in other studies [5,82].

Since qPCR results indicated no decrease in bacterial abundance, an increase in relative proportions might indicate an increase in abundance. The *Pseudarcicella* lineage increased from 1.82% downstream of the Jaune River to 17.89% at the DWTP, 9.8 km downstream (Figure 7), suggesting that an abundance of this lineage could have been multiplied 10 times (theoretical abundances according qPCR analyses: from 8.4 × 10^3^ 16S rRNA gene per mL of filtered water downstream of the Jaune River to 1.14 × 10^5^ genes per mL at the DWTP) along this part of the river. Considering a water travel time of approximately 3 h (L. Collin, pers. com.), this corresponds to a suspicious doubling rate of approximately 45 min in the exponential growth phase. However, organisms might travel downstream at a lesser mean velocity than the water in the main channel [76,83]. Indeed, rivers contain many areas of slow or non-flowing water, such as banks, eddies behind boulders, fallen trees, or blind arms, reducing the average velocity of the water body [84,85] and offering a possible mechanism for prolonging planktonic residence that allows the rise of lotic microbial populations such as *Pseudarcicella*/*Aquirula* species.

## 5. Conclusions

Despite the small variations in environmental conditions along the river transect, we observed progressive but important changes in both the bacterial and eukaryotic community structures. Our results reveal an ecological succession within connected freshwater ecosystems with a progressive replacement of microbial communities along the river course, leading to potential modifications of the water composition between lake outlets and rivers and supporting the River Continuum Concept [23], as previously demonstrated in some large rivers [15,24,25,26,27,28]. This drift in microbial community composition likely results from the shift from a stratified lentic to a turbulent ecosystem, whose differences are particularly marked in late summer (time of sampling). Microbial communities detected in the lake could be better adapted to an environment with steady conditions of light availability or nutrient concentrations. In the river, these communities could be negatively impacted by the water turbulence, leading to flocculation, the sedimentation of coarse particles, and variation in environmental conditions. The decrease of these populations in the water column of the river could allow the development of other microbial communities, emerging from the lake rare biosphere or inoculated by tributaries, meanders, or river sediments, which are more adapted to the turbulent river conditions. Other changes will probably occur downstream with the increase of populations with different lifestyle strategies as observed in other rivers along longer transects [27]. However, our results indicated that major changes in bacterial and eukaryotic communities can occur quickly in rivers (within the first 11 km of the river in the case presented herein) and might be favored by the meandering feature of the river, with potential consequences for biogeochemical cycles and carbon transformations.

## Supplementary Materials

The following are available online at https://www.mdpi.com/2076-2607/8/11/1631/s1, Figure S1: Detection pattern of bacterial and eukaryotic OTUs, Figure S2: Results of the similarity search with IMNGS server, Figure S3: Variations in relative proportions of selected OTUs and lineages along the water course of the Saint-Charles River, Figure S4: Bacterial 16S rRNA gene quantification along the water course of the Saint-Charles River.
