# Supplementary material for: Rapid Changes in Microbial Community Structures along a Meandering River
Source: Microorganisms. 2020 Oct 22;8(11):1631. doi: 10.3390/microorganisms8111631 (PMC7690413; doi:10.3390/microorganisms8111631)
Supplement: Supplementary file 1 [file microorganisms-08-01631-s001.zip › FiguresSup_SoTa_v15052020.pdf]

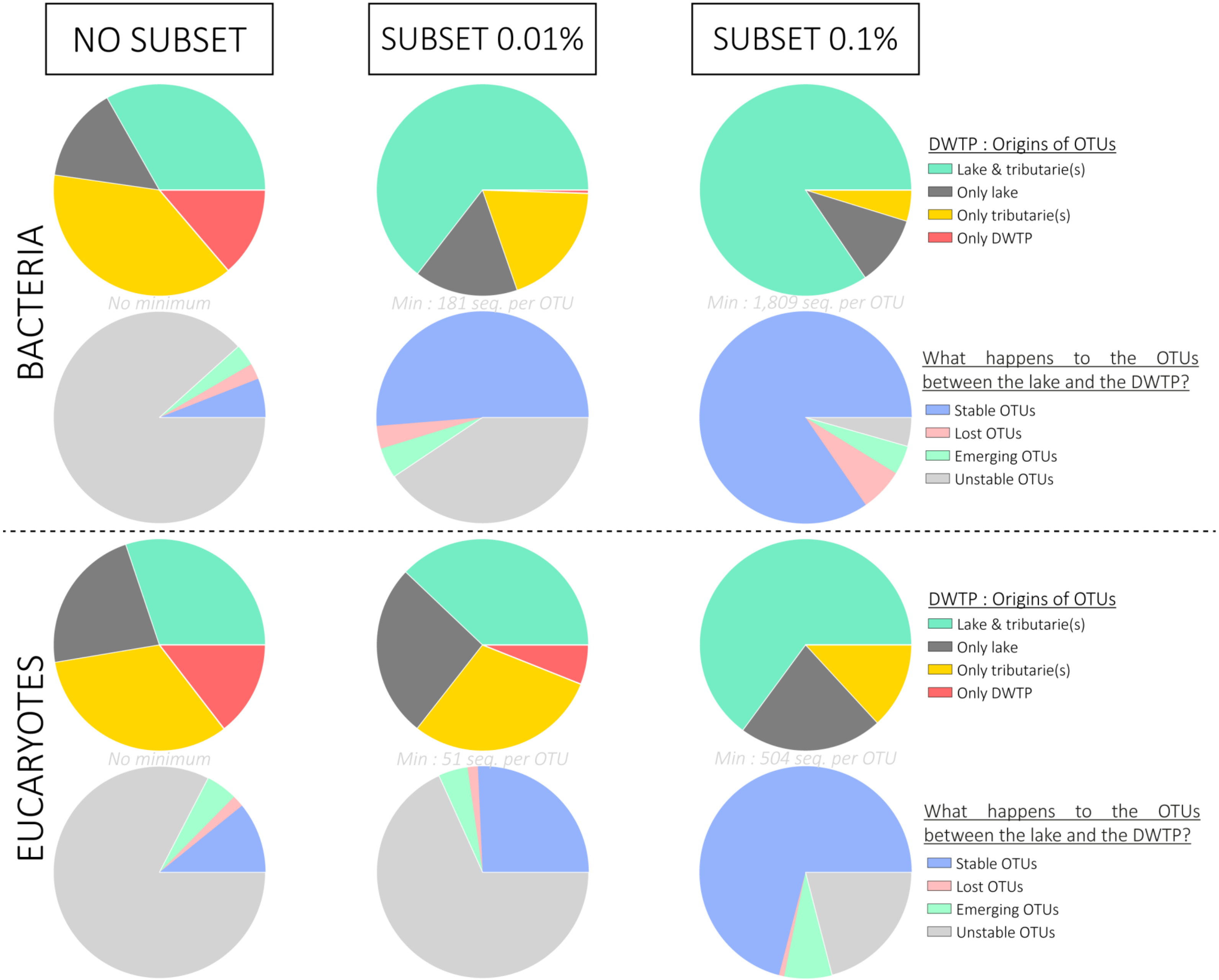


**<u>Figure S1</u>**: Detection pattern of bacterial (top) and eukaryotic (bottom) OTUs along the water course of the Saint-Charles River. Potential origins of bacterial OTUs detected at the DWTP sampling site (pie at the top, yellow/green/dark grey/red pies) and detection pattern of the OTUs along the water course of the Saint-Charles River (pie at the bottom, blue/red/green/gray pies).

## Samples containing the highest proportions of sequences sharing > 99% similarity with the sequence of the *Pseudarcicella* OTU

| Sample ID | Proportion of *Pseudarcicella* OTU (99% sim.) | Proportion of *Cyanobium* OTU (99% sim.) | Sampling site |
|---|---|---|---|
| SRR3113034 | 43,8% | 0,0% | Victoria, Australia |
| SRR3113037 | 36,9% | 0,0% | Victoria, Australia |
| SRR2077059 | 36,4% | 0,0% | British Columbia, Canada |
| SRR3112992 | 34,8% | 0,0% | Victoria, Australia |
| SRR6510452 | 34,6% | 0,0% | Maine, USA |
| SRR3112990 | 30,9% | 0,0% | Victoria, Australia |
| SRR3113036 | 28,4% | 0,0% | Victoria, Australia |
| SRR3112985 | 27,8% | 0,0% | Victoria, Australia |
| SRR3113021 | 25,9% | 0,0% | Victoria, Australia |
| SRR3112989 | 25,3% | 0,0% | Victoria, Australia |
| SRR3112988 | 24,3% | 0,0% | Victoria, Australia |
| SRR2076881 | 22,2% | 0,0% | British Columbia, Canada |
| SRR2063698 | 21,6% | 0,0% | Chicago, USA |
| SRR3598206 | 21,2% | 0,0% | Georgia, USA |
| SRR3597989 | 20,6% | 0,0% | Georgia, USA |
| SRR3112857 | 19,7% | 0,0% | Victoria, Australia |
| SRR3598122 | 19,1% | 0,0% | Georgia, USA |
| SRR3598154 | 18,8% | 0,0% | Georgia, USA |
| SRR6313930 | 18,7% | 0,0% | Xinjiang, China |
| SRR3598150 | 17,8% | 0,0% | Georgia, USA |
| SRR4102247 | 17,8% | 0,0% | Shannxi, China |
| SRR6510453 | 17,1% | 0,0% | Victoria, Australia |
| SRR3113040 | 16,7% | 0,0% | Victoria, Australia |
| SRR3598078 | 16,5% | 0,0% | Georgia, USA |
| SRR3598212 | 16,5% | 0,0% | Georgia, USA |
| SRR1620867 | 16,2% | 0,0% | Switzerland |
| SRR3597991 | 16,2% | 0,0% | Georgia, USA |
| SRR6243863 | 15,9% | 0,0% | Pyrenees, Spain |
| SRR1620874 | 15,8% | 0,0% | Switzerland |
| SRR6510409 | 15,3% | 0,0% | Victoria, Australia |

## Samples containing the highest proportions of sequences sharing > 99% similarity with the sequence of the *Cyanobium* PCC6307 OTU

| Sample ID | Proportion of *Pseudarcicella* OTU (99% sim.) | Proportion of *Cyanobium* OTU (99% sim.) | Sampling site |
|---|---|---|---|
| ERR1299040 | 0,1% | 12,3% | NY, USA |
| SRR6059092 | 0,0% | 11,6% | Arkhangelsk, Russia |
| SRR5460525 | 0,0% | 9,8% | Xiamen, China |
| SRR6417045 | 0,0% | 8,7% | Ningbo, China |
| SRR6417048 | 0,0% | 8,5% | Ningbo, China |
| SRR6059087 | 0,0% | 7,3% | Arkhangelsk, Russia |
| SRR6059093 | 0,0% | 6,9% | Arkhangelsk, Russia |
| SRR6059095 | 0,0% | 6,8% | Arkhangelsk, Russia |
| SRR6059094 | 0,0% | 6,5% | Arkhangelsk, Russia |
| SRR6417034 | 0,0% | 5,7% | Ningbo, China |
| SRR7081387 | 3,1% | 4,7% | Minessota, USA |
| SRR5460670 | 0,0% | 4,5% | Xiamen, China |
| SRR6243712 | 7,2% | 4,3% | Pyrenees, Spain |
| SRR6243914 | 0,8% | 3,6% | Pyrenees, Spain |
| ERR1299039 | 1,0% | 3,5% | NY, USA |
| SRR6059086 | 0,0% | 3,4% | Arkhangelsk, Russia |
| SRR6059090 | 0,3% | 3,4% | Arkhangelsk, Russia |
| SRR3598044 | 1,5% | 3,2% | Georgia, USA |
| ERR1377212 | 1,1% | 3,0% | China |
| SRR6417047 | 1,2% | 2,8% | Ningbo, China |
| SRR6059097 | 0,0% | 2,7% | Arkhangelsk, Russia |
| SRR5460746 | 0,0% | 2,3% | Xiamen, China |
| ERR1740142 | 0,3% | 2,3% | Quebec, Canada |
| SRR6059096 | 0,0% | 2,2% | Arkhangelsk, Russia |
| ERR1101412 | 0,3% | 2,0% | Quebec, Canada |
| SRR7081284 | 2,1% | 2,0% | Minessota, USA |
| ERR1377225 | 0,0% | 2,0% | China |
| ERR1377221 | 0,9% | 1,9% | China |
| SRR5460744 | 0,0% | 1,9% | Xiamen, China |
| ERR1377252 | 0,2% | 1,7% | China |

Type of sample
- River or stream
- Lake
- Unknown

**Figure S2**: . Results of the similarity search with the IMNGS server for two selected OTUs (OTU affiliated with *Pseudarcicella* on the left and *Cyanobium* PCC6307 on the right).

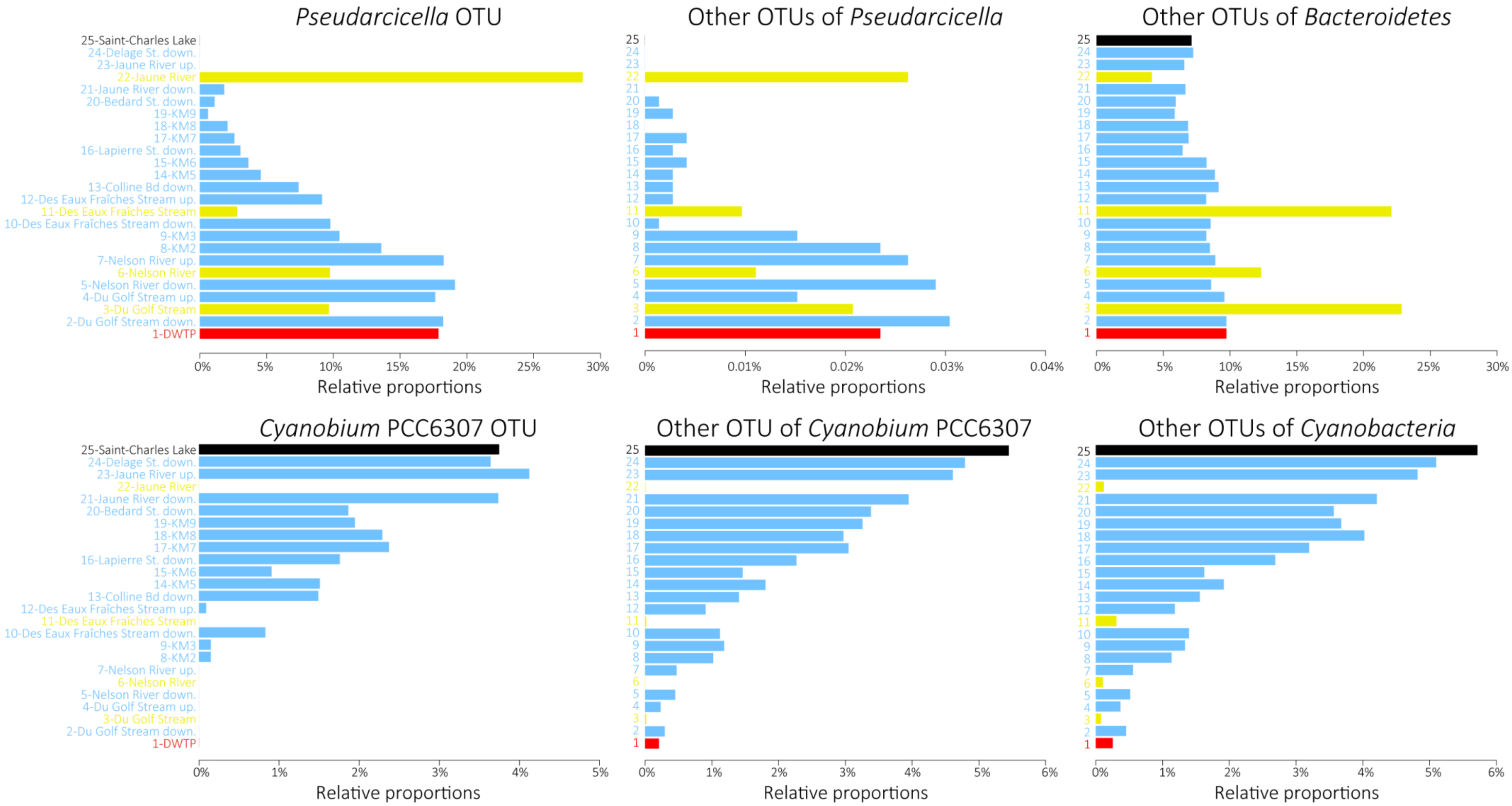


**Figure S3**: . Variations in relative proportions of selected OTUs and lineages along the water course of the Saint-Charles River. Top : Variations of the emerging *Pseudarcicella* OTU (left, cf. Figure 5), variations of the other OTUs affiliated with *Pseudarcicella* without the former (center) and variations of the *Bacteroidetes* phylum without the emerging *Pseudarcicella* OTU (right). Bottom : Variations of the lost *Cyanobium* PCC6307 OTU (left, cf. Figure 5), variations of the other OTUs affiliated with *Cyanobium* PCC6307 without the former (center) and variations of the *Cyanobacteria* phylum without the lost *Cyanobium* PCC6307 (right).

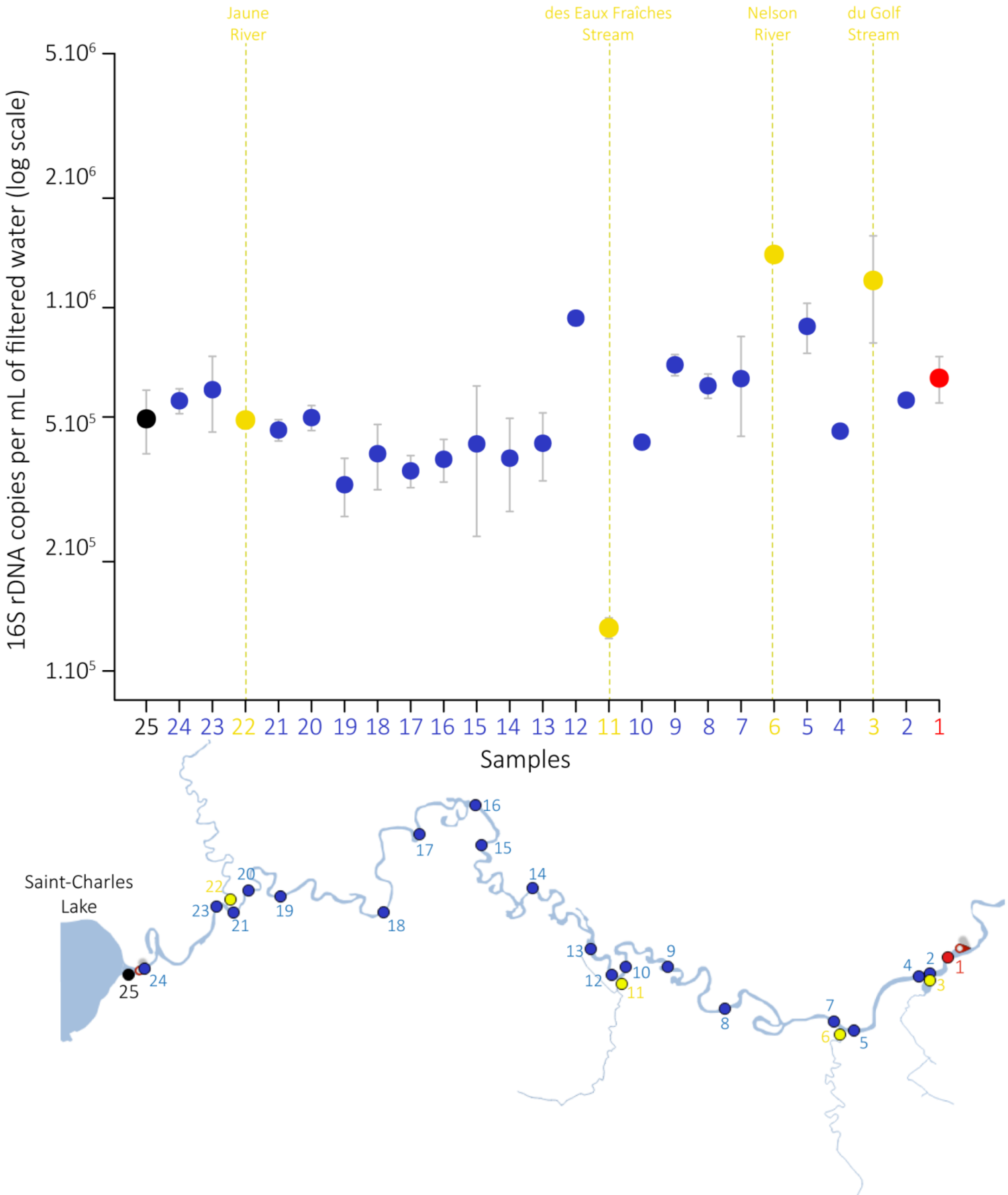


**Figure S4**: . Bacterial 16S rRNA gene quantification (qPCR) along the water course of the Saint-Charles River. Blue and red dots represent samples collected in the Saint-Charles River (red dot for the DWTP sampling point), yellow dots represent samples collected in the tributaries and black dot represent the water sample collected in the Saint-Charles Lake. The tributary locations are highlighted by the yellow dotted lines.
